# Prevalence, medication use, and health care utilization in pediatric primary headache: a school-based cross-sectional study

**DOI:** 10.1186/s10194-026-02299-x

**Published:** 2026-02-20

**Authors:** Mira Jaso, Allison M. Smith, Henrike Goldstein, Matthias Sehlbrede, Julia Wager

**Affiliations:** 1 Department of Children’s Pain Therapy and Paediatric Palliative Care, Faculty of Health, School of Medicine, Witten/Herdecke University, Witten, Germany; 2 Division of Pain Medicine, Department of Anesthesiology, Perioperative and Pain Medicine, Boston Children’s Hospital, Boston, MA USA; 3 Division of Psychology, Department of Psychiatry, Harvard Medical School, Boston, MA USA; 4 German Paediatric Pain Centre, Children’s and Adolescents’ Hospital Datteln, Datteln, Germany; 5 PedScience Research Institute, Datteln, Germany

**Keywords:** Pediatric, Epidemiology, Primary headache, Chronic headache, Medication use, Health care utilization, Migraine, Tension type headache

## Abstract

**Background:**

Primary headache disorders are the most common neurological conditions affecting children and adolescents and among the leading causes of disability worldwide. However, population-level data on prevalence, medication use, and health care utilization remain limited. This study assessed the prevalence of recurrent primary headache, migraine, and tension type headache, and examined associated patterns of medication use and health care utilization.

**Methods:**

A cross-sectional survey was conducted among students in grades 6–12 across 11 schools in Central Florida, USA. Self-administered questionnaires captured headache characteristics and relevant clinical, psychological, and behavioral factors. Migraine and tension type headache were classified according to self-reported International Classification of Headache Disorders, 3rd Edition diagnostic criteria. Descriptive statistics and regression models examined demographic associations with headache type and prediction models of medication use and health care utilization.

**Results:**

The sample included *N* = 583 students (*M*_age_*=*13.58, *SD =* 2.25), of whom 53% were female. Overall, 67% reported experiencing at least one headache in the past three months. In the total sample, 30% of students reported at least weekly recurrent primary headache, 18.2% fulfilled criteria for migraine and 24.9% for tension type headache. Female students had higher odds of experiencing recurrent primary headache than male students (OR = 2.80, 95% CI [1.90, 4.12]), while age was not a significant predictor. Among students experiencing recurrent primary headache, 73.7% reported using medication, and 24.6% had sought medical attention for headaches in the past three months. Over-the-counter medication use was predicted by higher headache frequency, passive pain coping, and social support seeking. Prescription medication use was predicted by sex, language, headache intensity and frequency, stress and passive pain coping strategies. No demographic, psychological or clinical variables significantly predicted headache-related health care utilization in this sample.

**Conclusions:**

Primary headaches and associated medication use are highly prevalent in youth. These findings highlight the need for improved monitoring and targeted intervention strategies to reduce the prevalence and burden of pediatric primary headache disorders. Increased attention to medication patterns and health care utilization may inform and facilitate action in health policy responses.

**Supplementary Information:**

The online version contains supplementary material available at 10.1186/s10194-026-02299-x.

## Background

Pediatric primary headache, headache with no known underlying pathologic cause, constitutes the most common neurological disorder and the most prevalent pain manifestation in children and adolescents worldwide [1, 2]. While varying study designs and methodologies complicate consistent reporting of prevalence, recent studies estimate prevalence ranges from 10% to 51% in younger children and 21% to 85% in adolescents [3–5]. Overall, prevalence of pediatric primary headache is higher in girls and older children [5–7]. Although prevalence estimates vary, recent global analyses indicate a substantial increase in pediatric migraine. Dong et al. [8] reported that both incidence and prevalence of pediatric migraine increased substantially from 1990 to 2019, with the highest burden observed among adolescents. In contrast, tension-type headache (TTH) has remained comparatively stable over time, with Yao et al. [9] noting only a slight increase in age-standardized prevalence of TTH among children and adolescents between 1990 and 2021. Despite these trends, pediatric headache epidemiology remains underrepresented in the scientific literature [3]. Consequently, early detection and adequate treatment are often lacking, contributing to considerable burdens on children, families, and the health care systems that serve them. Affected youth frequently experience impairments in academic performance, quality of life, psychological well-being, engagement in social and leisure activities, physical and cognitive functioning [10], as well as the potential and likely long-term chronification of these symptoms [11, 12].

The exact etiology of recurrent primary headache (RPHA) in children and adolescents remains unclear. While earlier research has emphasized biological mechanisms, newer evidence supports a biopsychosocial model of etiology [13], which highlights biological, psychological and social/environmental influences on the development, maintenance and progression of headache disorders [14]. Although widely accepted in chronic pain treatment, biomedical approaches persist as dominant in clinical practice concerning primary headache [13]. Pharmacologic interventions remain common [15], despite evidence that some medications may be ineffective, exacerbate headaches, or pose risks for dependency and withdrawal [16, 17]. Recent studies have found that between 49 and 65.3% of pediatric patients in Saudi Arabia, Austria, and Germany used over-the-counter (OTC) and prescription medications to alleviate primary headache [5, 18, 19]. High rates of medication use have been linked to headache chronification [20], underscoring the importance of understanding the predictors of headache-related medication use. Research also shows that health care utilization (HCU) is higher in youth exhibiting a more severe pain presentation [19]. Guided by clinical relevance and prior research, the present study focused on examining a set of key predictors of medication use and HCU in youth, including headache subtype (migraine and TTH), headache frequency, and associated functional disability [21], as well as demographic variables (e.g., age, sex) which have demonstrated influence on treatment medication use and HCU [22, 23]. Given prior evidence linking these factors to headaches, additional constructs explored here include language [24–26], self-efficacy and coping strategies [27, 28], sleep quality [29, 30], stress [31, 32], and pain intensity [19, 33].

The first aim of this study is to explore the overall prevalence of RPHA, including age- and sex-specific patterns of migraine and TTH. This provides valuable insight into current disease-related burden in pediatric populations. Furthermore, the second aim, identifying predictors of medication use and HCU in youth, may inform the development of tailored prevention strategies, improve headache management and support more efficient clinical resource allocation. Together, these two aims represent timely and clinically relevant directions for research efforts.

## Methods

### Study design

The present study utilized a cross-sectional school-based design, with data collection occurring in September and October 2024 across 11 middle and high schools in Central Florida, USA. School recruitment was limited to private and charter schools; access to public schools was not permitted due to district policy prohibiting the use of instructional time for external research activities. A convenience sampling method was employed, resulting in a final sample of nine private and two charter schools. This approach was selected to maximize participation through focus on schools that were accessible and willing to participate within the data collection period. As a result, convenience sampling enabled the research team to efficiently recruit a large sample within the practical limitations of time and accessibility.

### Procedure

The study recruited all sixth through twelfth grade students who provided signed parental consent and student assent, and who possessed sufficient English language proficiency to understand the study materials as participants. Once schools had expressed agreement to participate, both students and their parents or guardians were provided with comprehensive verbal and written information outlining the study’s procedures, confidentiality measures, and research objectives to ensure clarity and understanding. Additionally, parents and guardians were invited to attend either in-person or virtual presentations about the study, conducted by the primary researcher, to further promote support and participation. Depending on the available technological resources at each school, participants completed the questionnaire either electronically, via a secure online platform (LimeSurvey), or using a paper-pencil format. Questionnaires were administered during regular school hours in a supervised classroom setting. Participants were given up to 45 min to complete the materials. Instructions were provided verbally, and the primary researcher was available to answer questions. Absent students were given the opportunity to participate at a later date to minimize selection bias. All responses were collected anonymously. To maintain confidentiality and ensure that each student participated only once, participants created a self-generated identification code (SGIC) by answering six salient, non-identifying personal questions (see AF1 in additional files).

### Sample

A total of 15 schools were approached, of which 11 agreed to partake in this study, providing access to a student population of 2,305 youth. Of this group, *N* = 646 students (28%) were successfully recruited to participate. At the school with the largest target population (1,000 students), researchers were not permitted on campus due to security restrictions, resulting in a low recruitment rate (2%). Across the remaining 10 schools, student participation rates ranged from 16% to 91% with an average rate of 59%. The lowest of these (16%) occurred at a high school where the majority of students independently managed their transportation, thereby reducing direct access to parents, limiting consent distribution and impeding parental follow-up. The following participant exclusions were applied: four students were excluded for exceeding the upper age limit of 19 years, one participant was removed at parental request, and 58 students submitted entirely blank questionnaires or were absent during all data collection attempts. As a result, the final analytic sample consisted of *N* = 583 participants.

### Measures

Participants completed a series of demographic questions, including age, grade, sex (i.e., male, female, prefer not to answer), race, ethnicity, and the language they first learned to speak. Race was reported using standardized categories (i.e., American Indian or Alaska Native, East Asian, South Asian, Black or African American, Native Hawaiian or other Pacific Islander, White, Other, or Unknown), and ethnicity was classified as Hispanic or Latino, not Hispanic or Latino, or Unknown. Participants could select multiple race categories. Primary language was assessed using a forced choice format (e.g., English, Spanish, French, Portuguese, Arabic, or Other). Measures varied in their reporting time frames. PROMIS instruments assessed sleep and stress over the prior week to capture current symptomology and reduce recall bias, whereas headache characteristics were assessed over the preceding three months to characterize typical headache patterns. This distinction is particularly important for children with migraines, as attacks may occur infrequently [34, 35], making a longer recall window necessary to capture representative headache patterns. Importantly, perceived stress and sleep disturbance demonstrate adequate short-term stability, with PROMIS measures showing good test-retest reliability [36, 37], supporting their use as indicators of relatively stable vulnerability rather than short term states.

#### Headache assessment

The prevalence of RPHA was assessed through participant retrospective self-report and defined as headache occurring at least weekly with first headache onset more than three months prior to questionnaire completion. Participants who reported headache onset within the past three months were excluded from subsequent RPHA subgroup analyses. Headache subtype assessment was conducted using a structured diagnostic questionnaire based on the International Classification of Headache Disorders, 3rd edition (ICHD-3) diagnostic criteria, with items designed to distinguish between migraine and TTH.

##### Frequency

Participants who reported experiencing headaches in the past three months were asked to indicate the number of headache days during this time period (ranging from 1 to 90) and whether they typically experienced one or more headache days per week (yes/no).

##### Onset and duration

Participants were asked to indicate headache onset (less than 3 months ago, 3–6 months ago, 6–12 months ago, 1–2 years ago, over 2 years ago) and how long their headaches typically last (less than 1 h, 1–2 h, 2 h up to 1 day, 1–3 days, 4 days or longer).

##### Pain intensity

Participants rated their “usual” and “worst” headache intensity on 11-point numerical rating scales (NRS), ranging from 0 = no pain at all to 10 = worst/strongest pain.

##### Pain quality

Participants indicated whether their headache pain was pulsating, pressing or tightening like a tight bike helmet, or pulsating at times and pressing or tightening at other times.

##### Location

Participants reported the location of their headache pain by selecting one of the following options: “on both sides of your head”, “only on one side of your head”, “sometimes on both sides of your head and sometimes only on one side of your head”.

##### Additional symptoms

Participants reported additional symptoms experienced during headaches by selecting from the following options: nausea, vomiting, sensitivity to light or sound, unusual sensations in the hands, flickering vision, dizziness, and altered taste or smell. Participants also indicated the perceived impact of physical activity on their headaches by completing the sentence: “When I have a headache and I move or exercise…” with one of the following answer options: “…my headache usually gets better”, “…my headache usually gets worse”, “…my headache usually stays the same”, or “…my headache sometimes gets worse, other times it stays the same or gets better”.

##### Prior headache diagnosis

Participants were asked if they had ever received a formal headache diagnosis from a doctor (yes/no) and if so, what that diagnosis was, by selecting all applicable answer options: TTH, migraine, mixed headache syndrome, new daily persistent headache, post-concussive headache, post-infectious headache, and other.

#### Psychological factors

##### Coping with pain

Strategies for coping with headache were evaluated using the Pediatric Pain Coping Inventory-Revised (PPCIr), which includes subscales for passive pain coping (10 items, e.g., When I am in pain, I … ask for medication; $\:\alpha\:$ = 0.73), social support seeking (8 items, e.g., … I want to be held; $\:\alpha\:$ = 0.73), and positive self-instruction (7 items, e.g., … I encourage myself to be brave; $\:\alpha\:$ = 0.70). PPCIr items were rated on a three-point scale (0 = almost never, 1 = sometimes, 2 = often). Scores were summed for each subscale with higher scores reflecting more use of that type of coping strategy. For the purposes of this study, the phrase “When I am in pain, I…” was reworded to include headache “When I have a headache or I am in pain, I…”.

##### Pain self-efficacy

Participant perceived ability to function and manage pain was assessed with the 11-item Scale for Pain Self-Efficacy (SPaSE) [38] using a five-point Likert scale (ranging from 0 = not true to 4 = true; possible range: 0–44). Scores were summed, with higher scores reflecting higher pain-related self-efficacy. The internal consistency of the scale was $\:\alpha\:$ = 0.85.

##### Stress

Perceived stress was assessed using the four-item PROMIS Pediatric short form – Psychological Stress Experiences measure. This measure evaluated student stress over the past seven days using a five-point Likert scale (ranging from 1 = never to 5 = always). The internal consistency of the scale was $\:\alpha\:$ = 0.91.

#### Medication use

Participants were asked to indicate the frequency of headache-related medication use over the past three months, specifying the use of over-the-counter (OTC) medications and prescription rescue (RX) medications. OTC medication use in this study was defined as the use of single-ingredient analgesics, including acetaminophen (Tylenol) and NSAIDs such as ibuprofen (Motrin and Advil) and naproxen (Aleve). Combination products and OTC supplements were not included. Prescription rescue medications are treatments prescribed to relieve acute headache attacks once they begin, typically aiming to stop or reduce headache intensity and duration (e.g. triptans, ergotamines, or prescription-strength non-steroidal anti-inflammatory drugs [NSAIDs]). For both medication types, participants indicated whether they took the medication almost every time, sometimes, or never, when experiencing a headache. These categories were intended to reflect the relative frequency of medication use across headache episodes during the past three months, and no predefined cut-offs (e.g., percentage of treated episodes) were provided for participants. Participants also reported the number of times they had taken OTC and RX medications for headache relief over the past three months. In addition, participants reported the use of a daily preventative prescription (preventative RX) medication (reported as “yes/no”).

#### Health care utilization

Participants provided retrospective estimates of headache-related HCU in the past three months. Participants reported the type and frequency of headache-related health care visits, including consultations with primary care providers and/or specialists, urgent care centers, and emergency departments.

#### Additional measures

##### Functional disability

Participants completed the Functional Disability Inventory (FDI) [39], a 15-item measure assessing difficulty in performing physical and daily activities in home, school, recreational, and social domains over the past four weeks. Items were rated on a five-point Likert scale (ranging from 0 = no trouble to 4 = impossible), yielding a score from 0 to 60, with higher scores indicating greater pain-related functional impairment. The scale demonstrated good internal consistency in this sample ($\:\alpha\:$ = 0.87).

##### Sleep quality

Sleep quality was assessed using the eight-item PROMIS Sleep Disturbance short form, which evaluated different aspects of perceived sleep quality over the past seven days using several five-point Likert scales. Response options ranged from 1 = not at all to 5 = very much, 1 = never to 5 = always, and 1 = very poor to 5 = very good. Higher scores indicated increased sleep disturbance. The internal consistency of the scale in this sample was $\:\alpha\:$ = 0.89.

### Data analyses

Descriptive sample characteristics were provided as means (*M*) and standard deviations (*SD*), or number of participants (*n*) and percentages. The prevalence of RPHA and relevant primary headache subtypes (migraine and TTH) were examined in the full sample. Chi-square tests of independence were conducted to examine associations between the prevalence of RPHA, migraine, and TTH with participant sex. Cramer’s V was reported to interpret effect size. Odds ratios (ORs) were computed to quantify the strength of associations. Binary logistic regression analyses were performed to examine the association between age (entered as a continuous predictor) and the likelihood of experiencing RPHA, migraine, or TTH. For each model, the presence or absence of the respective headache subtype served as the binary dependent variable. Within the subsample of participants experiencing RPHA, we examined the frequency of headache-related OTC and RX medication use and HCU, including primary care providers/specialist visits, urgent care, and emergency department visits (all continuous variables). Incidence rate ratios (IRRs) were estimated to quantify the relative change in the expected frequency of medication use and HCU associated with each predictor.

#### Prediction models

##### OTC medication use

To explore potential associations between individual factors and the frequency of OTC medication use among students with RPHA, a series of negative binomial regression models were conducted. Each model included one predictor of interest: sex (female/male), age, language background (English/Non-English), migraine (yes/no), TTH (yes/no), headache frequency, pain intensity, functional disability (FDI), pain-related self-efficacy (SPaSE), coping strategies (PPCIr – passive pain coping, social support seeking, positive self-instruction), sleep quality (PROMIS SF v1.0 – Sleep Disturbance 8a), and stress (PROMIS SF v1.0 – Psychological Stress Experience 4a).

Subsequent multivariable analysis with all above mentioned variables was performed using negative binomial regression with a LASSO penalty in R [40]. Optimal model was selected based on 10-fold cross-validation. Individual predictors were tested for significance considering the selection during the LASSO regression [41]. Assumptions of multivariable models were assessed, and no violations were identified, as indicated by variance inflation factors (VIF $\:\le\:$ 2) and low bivariable correlations (*r* = $\:<$ 0.5).

##### RX medication use and HCU

The frequency of both RX medication use and HCU exhibited substantial zero inflation and overdispersion, warranting the use of zero-inflated negative binomial (ZINB) regression analyses to examine associations among students experiencing RPHA. Each model included one predictor of interest (see predictor list for OTC medication use analyses). The ZINB models allowed for the simultaneous estimation of predictors related to both the frequency of RX medication use and HCU (count component) and the likelihood of reporting zero RX medication use and HCU (logit component). The logit components are not relevant for the interpretation of results and are therefore reported in additional files (AF3 and AF4). Because zero-inflated models combine separate likelihood functions for the zero-inflation and count components, standard LASSO or other penalized regression methods that assume a single likelihood function cannot be directly applied for coefficient penalization across both components.

All analyses were conducted using IBM SPSS Statistics (version 29.0 for Mac), and R (Version 4.5.1; R Core Team, 2025). Utilized R packages: mpath [40], MASS [42], sjPlot [43], performance [44] and pscl [45]. Statistical significance was set at the conventional *p* $\:<$ 0.05 level for all analyses (two-tailed).

## Results

### Sample characteristics

Participant age ranged from 10 to 19 years (*M* = 13.58, *SD* = 2.25) with the sex distribution as follows: 52.7% female, 44.6% male, and 2.7% preferred not to disclose their sex. The prefer not to answer category (2.7%, *n* = 16) was coded as missing for any subsequent analyses computed by sex due to its small sample size. Detailed sample demographics are provided in Table 1.

**Table 1:** Descriptive statistics for sex, age, grade, race and ethnicity (*N* = 583)

Demographics		*N*	%	Mean	SD
Sex	Female	307	52.7		
	Male	260	44.6		
	Prefer not to answer	16	2.7		
Age	Range 10–19	583		13.58	2.24
Grade (Age)	6th (10–12)	136	23.3		
	7th (11–13)	122	20.9		
	8th (12–14)	110	18.9		
	9th (13–15)	34	5.8		
	10th (14–16)	49	8.4		
	11th (15–17)	47	8.1		
	12th (16–19)	85	14.6		
Race	American Indian/Alaskan Native	16	2.7		
	Black or African American	138	23.7		
	East Asian	27	4.6		
	Native Hawaiian or Pacific Islander	20	3.4		
	Other or Unknown	203	34.8		
	South Asian	27	4.6		
	White	286	49.1		
Ethnicity	Hispanic or Latino	217	37.2		
	Not Hispanic or Latino	267	45.8		
	Unknown	99	17.0		

### Prevalence of recurrent primary headache and headache subtypes

Overall, 67.0% of all participating students reported experiencing at least one headache in the past three months. The overall prevalence of RPHA in this school-based sample was 30.0%, with *n* = 175 students reporting at least weekly headaches for the previous three months and headache onset occurring at least three months prior to data collection. Among all participating students, 18.2% (*n* = 106) met criteria for migraine and 24.9% (*n* = 145) met criteria for TTH.

Among those experiencing RPHA, 35.4% (*n* = 62) met criteria for migraine and 29.7% (*n* = 52) met criteria for TTH only. Further, 34.9% of students (*n* = 61) did not meet diagnostic criteria for either migraine or TTH. No students met diagnostic criteria for both headache subtypes. Among students experiencing RPHA, only 18.9% (*n* = 33) reported having received a prior formal headache diagnosis from a physician. Of these, 57.6% (*n =* 19) indicated a migraine diagnosis, 33% (*n* = 11) reported TTH, 6.1% (*n* = 2) reported new daily persistent headache, and 3% (*n* = 1) reported a chronic migraine diagnosis. Additional response options, including *mixed headache syndrome*,* post-concussive headache*,* post-infectious headache*, and *I don’t remember*, were not selected.

In the following section, TTH and migraine classification is based on participant self-reported symptoms rather than prior physician diagnoses.

### Prevalence by sex

#### RPHA

A significant difference in prevalence of RPHA was observed by sex, χ²(1, *N* = 567) = 28.37, *p* $\:<$ 0.001. The effect was small to moderate (Cramér’s *V* = 0.22). RPHA was reported by 39.4% of female students compared to 18.8% of male students. The odds of experiencing RPHA were 2.8 times higher for female students compared to male students, *OR* = 2.80, 95% CI [1.90, 4.12].

#### Migraine

An association was found between sex and migraine prevalence, χ²(1, *N* = 567) = 16.57, *p* $\:<$ 0.001. Cramér’s V indicated a small to moderate effect (*V* = 0.17). Migraine was reported by 24.4% of female students and 11.2% of male students. The odds of experiencing migraine were 2.58 times greater for female students than for male students *OR* = 2.58, 95% CI [1.62, 4.10].

#### TTH

A chi-square test examining the association between sex and the prevalence of tension-type headache did not reach statistical significance, χ²(1, *N* = 567) = 0.35, *p* = 0.56. TTH was reported by 26.4% of female students and 24.2% of male students. Female students showed no higher odds of experiencing TTH than male students, *OR* = 1.12, 95% CI [0.77, 1.64].

Full prevalence data by sex are provided in additional files (AF2).

### Prevalence by age

**Fig. 1:**
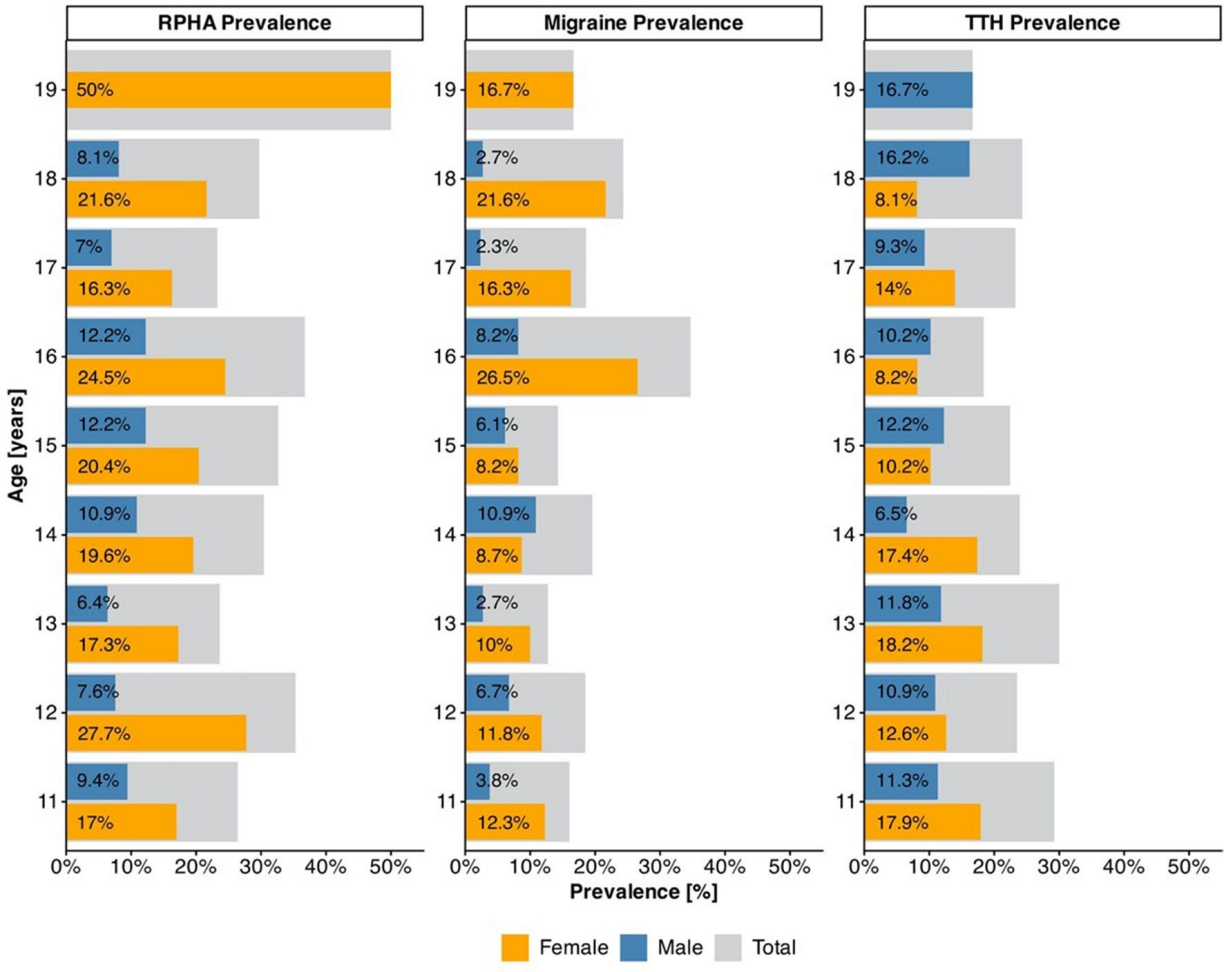
Recurrent primary headache and headache subtype prevalence by age. Note: *N* = 583; *n* = 16 students preferred not to disclose their sex and are thus not included in Fig. 1. Migraine and TTH categories include participants that did and did not meet criteria for RPHA

Fig. 1 presents the prevalence of RPHA and the two headache subtypes (migraine, TTH) by age and sex

Age did not significantly predict RPHA **(**OR = 1.004, 95% CI [0.93, 1.09]), migraine (OR = 1.086, 95%CI [0.99, 1.19]), or TTH (OR = 0.954, 95% CI [0.88, 1.04]).

### Medication use – prevalence and prediction model

#### Prevalence

In the full sample, 46.7% of students reported using medication in the preceding three months, regardless of headache status. To more closely examine medication use among high burden youth, we analyzed the subsample of students experiencing RPHA (*n* = 175). Medication use was defined as the consumption of OTC medications, preventative RX medications, and/or rescue RX medications to alleviate and/or manage headache-related pain. Overall, 73.7% of students (*n* = 129) experiencing RPHA reported using one or more types of medication to alleviate and/or manage headache-related pain in the preceding three months. Detailed frequencies of medication use by subtype are presented in Table 2.

**Table 2:** Prevalence of headache-related medication use (OTC, preventative RX, rescue RX)

Medication type	Frequency of Use per Headache Episode	Total (*n* = 175)	Frequency of Use in the Past 3 Months Median (IQR)
OTC	Yes	126 (72.4%)	3 (2–10) times
	Sometimes	88 (50.6%)	
	Almost every time	38 (21.8%)	
Rescue RX	Yes	26 (14.9%)	3 (1–10) times
	Sometimes	17 (9.8%)	
	Almost every time	9 (5.2%)	
Preventative RX	Yes	12 (6.9%)	

Note: Analyses based on subsample of students experiencing RPHA, *N* = 175

Detailed frequencies of medication use and medication subtype by grade (AF5), age (AF6), and sex (AF7) can be found in additional files.

### Prediction model – OTC medication use

#### Univariable analyses

Age, sex, self-reported migraine symptoms, self-reported TTH symptoms, headache intensity, headache frequency, self-efficacy, sleep quality, stress, and use of passive pain coping, social support seeking, and positive self-instruction strategies, were significantly associated with the frequency of OTC medication use in univariable regression analyses (see Table 3 for detailed results).

#### Multivariable model

After predictor selection through LASSO, a negative binomial regression model retained higher headache frequency (IRR = 1.02, 95% CI [1.01, 1.03]), passive pain coping strategies (IRR = 3.58, 95% CI [1.92, 6.70]), and social support seeking coping strategies (IRR = 2.22, 95% CI [1.16, 4.50]), as significant predictors of OTC medication use in students experiencing RPHA. Although age (IRR = 1.08, 95% CI [0.98, 1.19]) and self-reported migraine symptoms (IRR = 1.37, 95% CI [0.86–2.21]) were retained in the model, they did not reach statistical significance. *R*^*2*^ for the multivariable model was 37.6%.

**Table 3:** Factors associated with OTC medication use in youth experiencing RPHA

Predictors	Univariable analyses				Multivariable analyses		
	*n*	IRR	95%CI	*p*	IRR	95%CI	*p*
**Demographics**							
Age	175	1.098	1.023–1.177	0.009	1.076	0.975–1.192	0.136
Sex (Female)	170	1.968	1.377–2.813	< 0.001			
Language	175	1.455	0.995–2.127	0.053			
**HA characteristics**							
Migraine	175	1.969	1.423–2.726	< 0.001	1.373	0.864–2.208	0.185
TTH	175	0.562	0.397–0.795	0.001			
HA intensity	173	1.213	1.116–1.319	< 0.001			
HA frequency	174	1.020	1.013–1.027	< 0.001	1.016	1.006–1.026	< 0.001
**Psychological characteristics**							
Self-efficacy	173	0.973	0.955–0.992	0.006			
Sleep quality	170	0.963	0.963–0.981	< 0.001			
Stress	170	1.025	1.007–1.043	0.005			
**Coping Strategies**							
PPC	172	5.351	3.434–8.338	< 0.001	3.580	1.921–6.697	< 0.001
SSK	172	2.447	1.501–3.989	< 0.001	2.217	1.160–4.455	0.015
PSI	172	1.567	1.039–2.363	0.032			
Functional disability	175	1.020	1.000-1.040	0.053			

Note: PPC: Passive pain coping; SSK: Social support seeking; PSI: Positive self-instruction; HA: Headache; IRR: Incidence rate ratios

Headache subtype (migraine and TTH) was assigned using algorithms based on participant self-reported headache characteristics

Multivariable analysis conducted with complete data on all included variables (*n* = 164)

### Prediction model – RX medication use

Table 4 presents count component results of the ZINB regression examining predictors of RX medication use among children and adolescents experiencing RPHA. Female sex (IRR = 3.34, 95% CI [1.04–10.67]), greater headache intensity (IRR = 1.37, 95% CI [1.03–1.80]), higher headache frequency (IRR = 1.02, 95% CI [1.00-1.03]), greater perceived stress (IRR = 1.05, 95% CI [1.00-1.10]), and higher passive pain coping strategies (IRR = 14.06, 95% CI [1.11-177.53]) were each significantly associated with increased RX medication use. In contrast, youth who first learned to speak a language other than English demonstrated lower RX medication use (IRR = 0.32, 95% CI [0.12–0.85]). Detailed results from the logit component of the model are available in additional files (AF3).

**Table 4:** Factors associated with RX medication use in youth experiencing RPHA ZINB regression count component

Predictors	*n*	IRR	95% CI	*p*
**Demographics**				
Age	164	1.033	0.805–1.325	0.798
Sex (Female)	164	3.338	1.043–10.686	0.042
Language	164	0.322	0.122–0.847	0.022
**HA characteristics**				
Migraine	164	0.964	0.344–2.705	0.944
TTH	164	1.190	0.258–5.478	0.824
HA intensity	164	1.365	1.034–1.801	0.028
HA frequency	164	1.018	1.001–1.034	0.036
**Psychological characteristics**				
Self-efficacy	164	0.996	0.939–1.057	0.899
Sleep quality	164	0.974	0.920–1.032	0.375
Stress	164	1.050	1.003–1.099	0.035
**Coping Strategies**				
PPC^1^	164	14.056	1.113–177.5	0.041
SSK	164	1.215	0.297–4.970	0.787
PSI	164	1.834	0.656–5.132	0.248
Functional disability	164	1.043	0.980–1.110	0.184

Note: Abbreviations: PPC: Passive pain coping; SSK: Social support seeking; PSI: Positive self-instruction; HA: Headache; IRR: Incidence rate ratios; ZINB: zero inflated negative binomial

^1^Parameter estimates are unstable due to sample size restrictions resulting in very large IRRs and wide CI

Headache subtype (migraine and TTH) was assigned using algorithms based on participant self-reported headache characteristics

### Health care utilization – prevalence and prediction model

To analyze the prevalence of HCU, we examined the subsample of students experiencing RPHA (*n* = 175). Headache-related HCU was defined as seeking medical attention for headaches from primary care providers, specialists, as well as visits to urgent care centers or emergency departments within the past three months.

Reported overall HCU frequency across provider subtype ranged from 0 to 12 visits. Overall, 24.6% (*n* = 43) of students with RPHA reported seeking medical attention for headaches in the three months preceding data collection. Among these, the majority consulted primary care providers and/or specialists (*n* = 42; 24%), while 5.7% (*n* = 10) of students visited an urgent care center and 3.4% (*n* = 6) of students sought care in an emergency department. Of students reporting RPHA, 6.3% (*n* = 11) visited more than one provider subtype during this period. Table 5 provides detailed results for the prevalence of headache-related HCU, including subtypes, among students with RPHA.

**Table 5:** Prevalence of headache-related HCU in the past three months among students with RPHA

Type of HCU	*n*	%	Total visits	Mean (SD)	Range
PCP and/or specialist^1^	42	24%	94	0.54 (1.47)	0–10
Urgent care visits^2^	10	5.7%	17	0.10 (0.44)	0–3
ED visits^2^	6	3.4%	6	0.03 (0.18)	0–1

Note: HCU: Health care utilization, PCP: Primary care provider, ED: Emergency department, Percentages are based on the total number of students with RPHA, ^1^*N* = 175, ^2^*N* = 174

Detailed frequencies of HCU by sex and age can be found in additional files (AF8)

#### Prediction model – HCU

ZINB regression examined factors associated with HCU among children and adolescents experiencing RPHA. Demographic variables (age, sex, language), headache characteristics (migraine, TTH, headache intensity and frequency), psychological characteristics (functional impairment, self-efficacy, sleep quality, stress), and coping strategies were not significant predictors of HCU (all *p* > .05). Detailed results of the model are available in additional files (AF4).

## Discussion

This study provides a comprehensive analysis of the prevalence of RPHA and primary headache subtypes (migraine and TTH), as well as the prevalence, patterns, and predictors of medication use and HCU in a school-based sample of children and adolescents. Findings extend existing knowledge on the demographic, clinical, and psychological factors associated with medication use and HCU, with implications for early identification, intervention, and policy planning.

### Prevalence and demographic differences

Headache was highly prevalent in this U.S. school-based sample, with about two thirds of students reporting at least one headache over the preceding three months, and nearly one third meeting criteria for RPHA (at minimum, one headache per week for three or more months). Female students were more likely than male students to experience RPHA and migraine, consistent with prior evidence suggesting that sex-specific biological and psychosocial factors, including hormonal changes and differential stress responses, contribute to heightened headache vulnerability in adolescent biological females [2, 46]. In contrast, TTH prevalence did not differ significantly by sex, which may reflect relatively uniform exposure to contributing factors across biological sexes. Unlike migraine, TTH appears to be less influenced by hormonal fluctuations and sex-related differences in stress reactivity, which may contribute to fewer sex-based disparities [47].

Age was not significantly associated with RPHA, migraine, or TTH in the present sample. While some isolated findings similarly report no age-related differences in headache prevalence (e.g. [48]), this finding contrasts with numerous prior reports demonstrating higher headache prevalence among older adolescents [2, 5, 7, 49]. Comparisons with a German study indicate that younger grades in the present sample exhibited higher prevalence rates (e.g., 26% vs. 39% among 7th graders), potentially reflecting developmental or biological factors influencing headache patterns. For example, overall trends towards earlier menarche in U.S. populations [50] may contribute to relatively high prevalence among younger adolescents and reduce observable age differences. The absence of an age effect may also reflect cohort or methodological differences. For example, Philipp and colleagues [5] reported substantially higher *overall* headache prevalence among older adolescents (e.g., 80% among 11th graders) than observed in the present sample (68%) and Wager and colleagues [7] found higher prevalence of RPHA in older grades compared to the present sample (e.g., 41% vs. 26% among 10th graders). Variations in study design, including recall period for self-report and operational definitions of headache as well as sample characteristics, may partly account for these differences. Future research should examine how developmental, methodological, and biological factors interact to clarify mixed evidence regarding age effects in adolescents.

Despite the high headache burden associated with RPHA, only 18.9% of students reporting symptoms consistent with RPHA reported carrying a prior formal headache diagnosis. This diagnostic gap suggests that a number of clinically relevant cases may remain undiagnosed or untreated as specific headache disorders, possibly due to normalization or minimization of headache symptoms and/or limited health care access. These findings emphasize the need for proactive screening and early management strategies in pediatric and school health settings. Taken together, these prevalence patterns highlight the substantial burden of headache in U.S. youth and provide a critical foundation for understanding subsequent medication use and HCU patterns.

### Prevalence of overall medication use

In the full sample, nearly half of students (regardless of headache status) reported medication use in the preceding three months, closely aligning with community-based estimates from Denmark (43.6%; [51]), and Spain (42.2%;). Among youth with RPHA, 73.7% reported using at least one type of medication to manage or prevent headache-related pain within the preceding three months. OTC medications were most frequently used (72.4%), followed by RX rescue medications (14.9%), and preventative prescriptions (6.9%). These findings suggest strong reliance on self-directed OTC management and relatively limited engagement with prescription treatments, potentially reflecting barriers to accessing specialized care or limited awareness of potentially helpful pharmacologic management strategies. Comparable rates of medication use have been documented in chronic pain populations, such as Könning and colleagues [19], who reported that 65.3% of adolescents with chronic pain had used OTC pain medication in the preceding three months in a German school sample. In contrast, the medication use rates in the current study were substantially higher than those reported by further studies from Europe on chronic pain in youth. Specifically, Du and colleagues [52, 53] found that only 10-17.5% of German youth with chronic/recurrent pain used pain medication, while Perquin and colleagues [54] reported a rate of 39% among youth in the Netherlands. High rates of medication use among youth with headache, including risks for medication overuse, are well-documented in the literature [55, 56]. However, additional research is warranted to understand whether youth with headache use pain medication more frequently than youth with other chronic pain conditions, highlighting the need for further study. These discrepancies further underline the importance of examining whether differences in health care access, prescribing practices, or cultural norms surrounding medication use are associated with varying rates across populations and settings.

### Predictors of OTC medication use

In the multivariable model, higher headache frequency, greater use of passive pain coping strategies, and social support seeking strategies significantly predicted increased OTC medication use. With regard to headache frequency, each additional headache episode in the past three months was associated with a 1.6% increase in the rate of OTC medication use. Although causal inference cannot be drawn from this cross-sectional design, this pattern aligns with prior findings linking headache frequency to increased medication use [57]. Further, frequent OTC medication overuse has also been associated with increased headache frequency and may contribute to headache chronification [58]. These results highlight the clinical importance of monitoring self-medication behaviors and ensuring physician oversight to avoid inadvertent worsening of symptoms. Students reporting higher levels of passive pain coping tended to use OTC medication more frequently and greater social support seeking was similarly associated with higher use. These patterns align with conceptualizations of medication as a passive pain management strategy [59]. Youth who do not understand their headaches or lack confidence in self-management may rely on passive approaches, including waiting for relief or seeking help from others, potentially resulting in caregivers providing medication more frequently. In contrast, active self-management strategies such as exercise, relaxation, or distraction, require youth to act directly to alleviate pain. Importantly, Goldstein and colleagues [60] demonstrated that their web-based headache-education program was associated with significant reductions in passive pain coping in school children, suggesting that increasing knowledge may shift coping patterns in a more adaptive direction. These findings highlight the potential value of developmentally tailored headache education that empower youth to develop active coping skills, providing alternatives to medication and fostering more effective self-management. The multivariable model additionally included the non-significant predictors age and migraine, collectively explaining 37.6% of the variance in OTC medication use and emphasizing the substantial influence of clinical (e.g., headache characteristics) and psychological factors (e.g., coping style) in shaping treatment behaviors.

### Predictors of RX medication use

Patterns of RX medication use showed some overlap with those observed for OTC medication use. Greater headache frequency was associated with higher use, with each additional headache in the past three months corresponding to a 1.8% increase in the rate of RX medication use. Students who relied more heavily on passive pain coping were also more likely to use RX medications, however, these estimates were unstable (reflected by a large coefficient and wide CI) and should be interpreted with caution. Additional predictors emerged that did not mirror the OTC model. Female students reported substantially higher use (more than three times as frequent) consistent with prior findings demonstrating higher headache-related disability and medication use among biological females [61, 62]. Higher pain intensity was also associated with increased RX medication use, with each one-point increase in pain intensity, corresponding with a 36.5% increase in the rate of expected use. Further, increased self-reported stress levels corresponded with higher use (5% per one-point increase), aligning with evidence that poor mental health, including stress-related symptoms, may exacerbate headache burden and influence treatment-seeking behavior such as medication use [51]. In contrast, students who first learned a language other than English demonstrated significantly lower odds of RX medications use, showing an expected 67% lower rate compared to those who reported learning English first. This disparity may reflect cultural or linguistic barriers associated with access to, or utilization of, prescription care [63]. Together these findings suggest that clinical and psychological factors, along with demographic and psychosocial influences such as sex, stress, and language background may be associated with patterns of RX medication use. However, imprecise estimates and model instability due to the small number of participants reporting RX medication use warrant cautious interpretation. Replication in larger samples with more robust representation of RX medication users is warranted to confirm these associations.

### Prevalence of health care utilization

Among youth experiencing RPHA, 24.6% sought medical care for headaches in the preceding three months, most commonly from a PCP and/or specialist (24%) with relatively few reporting urgent care (5.7%) or ED (3.4%) visits. The average number of medical care visits was low across HCU subtypes and the overall number of visits per student ranged from 0 to 12. These findings broadly align with prior research from Japan, which reports that only about 34% of adolescents with headache sought physician care [64]. Further, a recent scoping review by Könning and colleagues [65] mirrors this trend, illustrating that even in the presence of significant pain-related disability, many adolescents do not engage with medical services or adhere to prescribed pain regimens.

### Predictors of health care utilization

None of the child-level clinical or psychosocial predictors examined emerged as significant predictors of HCU in this sample. These findings suggest that symptom burden and individual characteristics alone may not drive care-seeking behaviors in youth with RPHA. While child-level factors may influence self-management behaviors such as medication use, HCU is likely shaped by a broader set of determinants. Prior work shows that socioeconomic and structural factors, including household income, health insurance status, and systemic inequities, are strongly associated with whether children access health care [66, 67]. Additionally, parental beliefs and sociodemographic factors may outweigh a child’s symptom burden in determining HCU [68]. Taken together, these findings suggest that HCU in pediatric headache may be influenced more by family- and system-level determinants than by child-level clinical characteristics alone. The absence of significant predictors in our sample should not be interpreted as evidence of the absence of clinically meaningful drivers but likely reflects unmeasured factors. Efforts to better understand and improve HCU should therefore focus on enhancing caregiver understanding and awareness while addressing the structural barriers and socioeconomic conditions that limit access to care.

### Implications

These findings carry several implications for caregivers, pediatric PCPs, and school health professionals. The high prevalence of undiagnosed RPHA, migraine, and TTH in this sample underscores the potential benefits of routine headache screening in pediatric and school-based health settings. Brief, validated screening tools and surveys may facilitate early detection and targeted intervention, by identifying prognostic factors and potentially improving the rate of recovery [69]. Early detection also provides opportunities to educate youth and families on evidence-based management strategies, including the potential risks associated with medication overuse.

The gap observed between headache burden and HCU highlights potential barriers to care in this sample, including the normalization of headache symptoms in adolescents, limited access to pediatric headache specialists and reliance on self-management. Addressing these barriers through targeted education and school-based interventions may help prevent headache chronification and ensure that youth receive care aligned with their clinical needs. Clinicians may assess coping styles and refer youth for psychological support when maladaptive coping patterns are identified, in order to prevent inadvertent headache chronification [70, 71]. In this sample, the significant associations between passive pain coping, social support seeking and OTC medication use point to potential intervention targets. Similarly, the observed association between headache frequency and increased OTC medication use suggests that initial headache tracking could help youth recognize patterns and seek timely care. Biological female participants in this sample reported higher headache burden, highlighting the need for targeted education and monitoring. Cognitive-behavioral interventions that promote active coping and increase self-efficacy may also help reduce over-reliance on pharmacologic self-management. Culturally and linguistically tailored education on headache management is also warranted, providing accessible, multilingual information about headache types, triggers, and treatment options can empower families and reduce inequities in care access.

Overall, these results emphasize the urgent need for systematic screening and multifaceted intervention strategies to reduce headache burden, promote adaptive coping, and improve health outcomes among children and adolescents.

### Strengths and limitations

This study’s strengths include a diverse, school-based sample, enhancing ecological validity and capturing youth who may be underrepresented in clinical research. Distinguishing between OTC and RX medication use, as well as HCU, provided a multifactorial understanding of how specific predictors were uniquely associated with each outcome. For example, some factors predicted the frequency of RX medication use but were not associated with OTC medication use, highlighting relationships that could have been obscured if all medication use had been combined into a single measure.

Limitations of this study include its reliance on self-report, which may introduce recall bias or social desirability effects [72, 73]. Further, headache subtype classification was based on self-reported ICHD-3 criteria rather than clinical evaluation. While this approach is common in large-scale epidemiological studies, it does not replace clinical diagnosis. Consequently, misclassification of headache subtypes is possible, as participants may under- or over-report symptoms or misunderstand diagnostic questions, potentially affecting prevalence estimates and observed associations. Future research incorporating clinical interviews could help clarify subtype-specific patterns. Additionally, headache-related medication use was assessed using qualitative response options intended to capture children’s general approach to medication use across headache episodes (i.e., routine versus selective use), in addition to separate items assessing absolute frequency of use. As a result, categories such as “sometimes” and “almost every time” may have been interpreted variably across participants. The cross-sectional design of the study also precludes causal conclusions (e.g., whether passive pain coping strategies lead to higher medication use or vice versa). Another limitation relates to how primary headache subtypes were classified due to construct operationalization through differing self-reported indicators (i.e., usual pain intensity for TTH diagnosis; worst pain intensity for migraine diagnosis). This approach may have led to under-identification of headache subtypes (TTH and migraine). Potential selection bias is also a concern, as the study relied on convenience sampling within schools. Students who were absent from school due to pain may have been systematically excluded, potentially underestimating the prevalence of RPHA, migraine, and/or TTH. Conversely, students experiencing headaches may have been more likely to obtain parental consent and provide assent to participate, potentially inflating prevalence estimates. Additionally, although two public charter schools were included in our sample, the exclusion of traditional public school settings may limit the generalizability of findings, particularly for students from the lowest socioeconomic strata.

Finally, small subgroup sizes, particularly for RX medication use and HCU, limited parameter stability in our models, resulting in wide confidence intervals and large incidence rate ratios for some predictors (e.g., passive pain coping). This limitation reduces the precision and interpretability of the models. Conclusions regarding predictors of RX medication use and HCU should therefore be interpreted cautiously. Replication in larger samples with more robust subgroup representation is warranted.

## Conclusion

This study offers a comprehensive, school-based analysis of recurrent primary headache, migraine, and tension-type headache among youth, documenting high prevalence, underdiagnosis, and differential predictors of OTC and RX medication use, and HCU. Findings underscore the widespread and chronic nature of primary headache in children and adolescents, the central role of coping behaviors in shaping medication use, and the persistent gap between headache burden and health care utilization (and in turn, potentially helpful clinical management). Interventions promoting early identification, culturally responsive care, and adaptive coping skills are needed to optimize headache outcomes for these youth. Longitudinal and intervention-based research should further clarify causal pathways and inform evidence-based policy and practice in pediatric headache care.

## Supplementary Information

Below is the link to the electronic supplementary material.

Supplementary Material 1

Supplementary Material 2

## Data Availability

The datasets generated and/or analyzed during the current study are not publicly available due to ethical and legal restrictions related to the inclusion of minor participants and the presence of protected health information. De-identified data are available from the corresponding author upon reasonable request and following IRB approval, where applicable.

## Abbreviations

RPHA: Recurrent primary headache
TTH: Tension type headache
HCU: Health care utilization
ICHD-3: International Classification of Headache Disorders, 3rd edition
OTC: Over-the-counter
RX: Prescription
SGIC: Self-generated identification code
NRS: Numerical rating scale
PPCIr: Pediatric Pain Coping Inventory - Revised
SPaSE: Scale for Pain Self-Efficacy
NSAID: Non-steroidal anti-inflammatory drug
FDI: Functional Disability Inventory
IRB: Institutional review board
M: Mean
SD: Standard deviation
IRR: Incidence rate ratio
ZINB: Zero-inflated negative binomial
CI: Confidence interval
PPC: Passive pain coping
SSK: Social support seeking
PSI: Positive self-instruction
PCP: Primary care provider
ED: Emergency department
