# Supplementary material for: Prevalence, medication use, and health care utilization in pediatric primary headache: a school-based cross-sectional study
Source: J Headache Pain. 2026 Feb 20;27(1):55. doi: 10.1186/s10194-026-02299-x (PMC12930800; doi:10.1186/s10194-026-02299-x)
Supplement: Supplementary file 2 — Supplementary Material 2 [file 10194_2026_2299_MOESM2_ESM.docx]

# Additional files

**AF1**

**Self-generated identification code questions**

|  | What’s the name of the month in which you were born? |
| --- | --- |
|  | What’s the FIRST letter of your MIDDLE name? If you don’t have one, just put 00. |
|  | Which school do you go to? |
|  | Which grade are you in? |
|  | Which sex were you assigned at birth? |
|  | IG/CG group randomization |

**AF2**

***Recurrent primary headache (RPHA) and headache subtype prevalence by sex (N = 583)***

|  | RPHA | Migraine | TTH |
| --- | --- | --- | --- |
| Sex |  |  |  |
| Female (*n* =307) | 121 (39.4%) | 75 (24.4%) | 81 (26.4%) |
| Male (*n* = 260) | 49 (18.8%) | 29 (11.2%) | 63 (24.2%) |
| PNTA (*n* = 16) | 5 (31.3%) | 2 (12.5%) | 1 (6.3%) |
| Total (*n* = 583) | 175 (30%) | 106 (18.2%) | 145 (24.9%) |

*Note:* Percentages are relative to each sex group i.e., 39.4% of participating female students experienced RPHA. PNTA: Prefer not to answer. Migraine and TTH categories include participants that did and did not meet criteria

for RPHA.

**AF3**

***Factors associated with RX medication use in youth experiencing RPHA***

***ZINB regression count component***

|  | **Count component (Neg Bin)** | | | | **Logit component (ZI)** | | |
| --- | --- | --- | --- | --- | --- | --- | --- |
| **Predictors** | ***n*** | **IRR** | **95%CI** | ***p*** | **OR** | **95%CI** | ***p*** |
| **Demographics** |  |  |  |  |  |  |  |
| Age | 164 | 1.033 | 0.805-1.325 | 0.798 | 1.060 | 0.863-1.302 | 0.580 |
| Sex (Female) | 164 | 3.338 | 1.043-10.686 | 0.042 | 0.716 | 0.240-2.141 | 0.550 |
| Language | 164 | 0.322 | 0.122-0.847 | 0.022 | 0.428 | 0.158-1.163 | 0.096 |
| **HA characteristics** |  |  |  |  |  |  |  |
| Migraine | 164 | 0.964 | 0.344-2.705 | 0.944 | 0.536 | 0.214-1.343 | 0.183 |
| TTH | 164 | 1.190 | 0.258-5.478 | 0.824 | 3.565 | 0.988-12.868 | 0.052 |
| HA intensity | 164 | 1.365 | 1.034-1.801 | 0.028 | 0.760 | 0.587-0.983 | 0.037 |
| HA frequency | 164 | 1.018 | 1.001-1.034 | 0.036 | 0.977 | 0.961-0.992 | 0.004 |
| **Psychological characteristics** |  |  |  |  |  |  |  |
| Self-efficacy | 164 | 0.996 | 0.939-1.057 | 0.899 | 1.050 | 0.998-1.104 | 0.059 |
| Sleep quality | 164 | 0.974 | 0.920-1.032 | 0.375 | 1.033 | 0.980-1.088 | 0.228 |
| Stress | 164 | 1.050 | 1.003-1.099 | 0.035 | 0.975 | 0.927-1.025 | 0.318 |
| **Coping Strategies** |  |  |  |  |  |  |  |
| PPC^1^ | 164 | 14.056 | 1.113-177.5 | 0.041 |  |  |  |
| SSK | 164 | 1.215 | 0.297-4.970 | 0.787 | 0.237 | 0.070-0.805 | 0.021 |
| PSI | 164 | 1.834 | 0.656-5.132 | 0.248 | 0.927 | 0.337-2.551 | 0.884 |
| Functional disability^1^ | 164 | 1.043 | 0.980-1.110 | 0.184 |  |  |  |

*Note:* Abbreviations: PPC: Passive pain coping; SSK: Social support seeking; PSI: Positive self-instruction; HA: Headache; IRR: Incidence rate ratios; ZI: zero inflated.

^1^Parameter estimates are unstable due to sample size restrictions resulting in very large IRRs and wide CI.

Headache subtype (migraine and TTH) was assigned using algorithms based on participant self-reported headache characteristics.

**AF4**

***Factors associated with HCU in youth experiencing RPHA***

***ZINB regression count component***

|  | **Count component (Neg Bin)** | | | | **Logit component (ZI)** | | |
| --- | --- | --- | --- | --- | --- | --- | --- |
| **Predictors** | ***n*** | **IRR** | **95%CI** | ***p*** | **OR** | **95%CI** | ***p*** |
| **Demographics** |  |  |  |  |  |  |  |
| Age^1^ | 162 | 0.905 | 0.736-1.112 | 0.340 |  |  |  |
| Sex (Female)^1^ | 162 | 0.685 | 0.284-1.655 | 0.401 |  |  |  |
| Language^1^ | 162 | 1.061 | 0.401-2.809 | 0.905 |  |  |  |
| **HA characteristics** |  |  |  |  |  |  |  |
| Migraine^1^ | 162 | 0.984 | 0.351-2.754 | 0.975 |  |  |  |
| TTH^1^ | 162 | 1.421 | 0.432-4.670 | 0.563 |  |  |  |
| HA intensity | 162 | 0.941 | 0.716-1.238 | 0.664 | 0.775 | 0.308-1.949 | 0.587 |
| HA frequency^1^ | 162 | 1.001 | 0.986-1.016 | 0.884 |  |  |  |
| **Psychological characteristics** |  |  |  |  |  |  |  |
| Self-efficacy | 162 | 0.993 | 0.947-1.040 | 0.754 | 1.433 | 0.665-3.089 | 0.358 |
| Sleep quality | 162 | 1.033 | 0.979-1.090 | 0.239 | 1.481 | 0.720-3.044 | 0.286 |
| Stress^1^ | 162 | 0.995 | 0.953-1.039 | 0.828 |  |  |  |
| **Coping Strategies** |  |  |  |  |  |  |  |
| PPC | 162 | 0.818 | 0.281-2.378 | 0.712 | 0.095 | 0.003-3.417 | 0.198 |
| SSK | 162 | 1.793 | 0.489-6.576 | 0.378 | 0.000 | 0.000-1587.1 | 0.247 |
| PSI | 162 | 0.889 | 0.337-2.346 | 0.812 | 0.000 | 0.000-8968.2 | 0.368 |
| Functional disability | 162 | 1.017 | 0.963-1.075 | 0.538 | 0.935 | 0.716-1.221 | 0.622 |

*Note:* Abbreviations: PPC: Passive pain coping; SSK: Social support seeking; PSI: Positive self-instruction; HA: Headache; IRR: Incidence rate ratios.

^1^Zero inflation (logit component) estimates are unstable due to sample size restrictions resulting in very large ORs and wide CIs.

Headache subtype (migraine and TTH) was assigned using algorithms based on participant self-reported headache characteristics.

**AF5**

***Prevalence of headache-related medication use (OTC, preventative RX, rescue RX) by grade***

|  | **Medication use** | |  |
| --- | --- | --- | --- |
|  | **OTC** (n = 127) | **Prev**. **Rx** (n = 13) | **Rescue Rx** (n = 26) |
| **Grade** |  |  |  |
| 6^th^ (n=36) | 24 (66.7%) | 3 (8.3%) | 5 (13.9%) |
| 7^th^ (n=47) | 33 (70.2%) | 2 (4.3%) | 8 (17%) |
| 8^th^ (n=29) | 19 (65.5%) | 2 (6.9%) | 5 (17.2%) |
| 9^th^ (n=5) | 5 (100%) | 1 (20%) | 0 |
| 10^th^ (n=13) | 9 (69.3%) | 0 | 2 (15.4%) |
| 11^th^ (n=18) | 15 (80.3%) | 3 (16.7%) | 2 (11.2%) |
| 12^th^ (n=27) | 22 (81.4%) | 2 (7.4%) | 4 (14.8%) |

*Note:* Analyses based on subsample of students with RPHA, *N* = 175

**AF6**

***Prevalence of headache-related medication use (OTC, preventative RX, rescue RX) by age***

|  | **Medication use** | |  |
| --- | --- | --- | --- |
|  | **OTC** | **Prev**. **Rx** | **Rescue Rx** |
| **Age** |  |  |  |
| 10 (n=2) | 2 (100%) | 0 | 1 (50%) |
| 11 (n=29) | 21 (72.4%) | 3 (10.3%) | 4 (13.8%) |
| 12 (n=45) | 29 (64.4%) | 2 (4.4%) | 6 (13.3%) |
| 13 (n=27) | 19 (70.4%) | 1 (3.7%) | 5 (18.52%) |
| 14 (n=14) | 10 (71.4%) | 2 (14.3%) | 2 (14.3%) |
| 15 (n=15) | 12 (80%) | 0 | 3 (20%) |
| 16 (n=18) | 14 (77.8%) | 2 (11.1%) | 1 (5.6%) |
| 17 (n=10) | 9 (90%) | 0 | 1 (10%) |
| 18 (n=11) | 9 (81.8%) | 1 (9.1%) | 3 (36.4%) |
| 19 (n=3) | 2 (66.7%) | 1 (33.3%) | 0 |

*Note:* Analyses based on subsample of students with RPHA, N = 175

**AF7**

***Prevalence of headache-related medication use (OTC, preventative RX, rescue RX) by sex***

| **Medication type** | **Frequency of Use** | **Female (*n* = 121)** | **Male (*n* = 49)** | **PNTA (*n* = 5)** | **Total (n = 175)** |
| --- | --- | --- | --- | --- | --- |
| **OTC** | Yes | 90 (74.4%) | 32 (65.3%) | 5 (100%) | 127 (72.6%) |
|  | Sometimes | 63 (52.1%) | 21 (42.9%) | 5 (100%) | 89 (50.9%) |
|  | Almost every time | 27 (22.3%) | 11 (22.4%) | 0 | 38 (21.7%) |
| **Rescue RX** | Yes | 20 (16.5%) | 6 (12.2%) | 0 | 26 (14.9%) |
|  | Sometimes | 14 (11.6%) | 3 (6.1%) | 0 | 17 (9.7%) |
|  | Almost every time | 6 (5%) | 3 (6.1%) | 0 | 9 (5.1%) |
| **Preventative RX** | Yes | 10 (8.3%) | 3 (6.1%) | 0 | 13 (7.4%) |

*Note:* Analyses based on subsample of students experiencing RPHA, *N* = 175

**AF8**

***Prevalence of headache-related health care utilization in students experiencing RPHA***

***by sex and age – N = 175***

|  | Health Care Utilization | | |
| --- | --- | --- | --- |
|  | PCP/SPEC | UC | ED |
| Sex |  |  |  |
| Female (n = 121) | 28 (23.1%) | 9 (7.5%) | 4 (3.3%) |
| Male (n = 49) | 13 (26.5%) | 1 (2%) | 2 (4.1%) |
| PNTA (n = 5) | 1 (20%) | 0 | 0 |
| Age |  |  |  |
| 10 (n = 2) | 0 | 0 | 0 |
| 11 (n = 29) | 7 (%) | 2 (%) | 1 (%) |
| 12 (n = 45) | 10 (%) | 3 (%) | 3 (%) |
| 13 (n = 27) | 10 (%) | 3 (%) | 1 (%) |
| 14 (n = 14) | 2 (%) | 0 | 0 |
| 15 (n = 16) | 4 (%) | 0 | 0 |
| 16 (n = 18) | 6 (%) | 1 (%) | 0 |
| 17 (n = 10) | 1 (%) | 0 | 0 |
| 18 (n = 11) | 1 (%) | 1 (%) | 1 (%) |
| 19 (n = 3) | 0 (%) | 0 | 0 |
|  | 42 | 10 | 6 |

*Note:* PCP: Primary care provider; SPEC: Specialist, UC: Urgent care, ED: Emergency department,

PNTA: Prefer not to answer; RPHA: Recurrent primary headache.

Row-percentages🡪 relative to sex and age totals
